# Unmet Clinical Need: Developing Prognostic Biomarkers and Precision Medicine to Forecast Early Tumor Relapse, Detect Chemo-Resistance and Improve Overall Survival in High-Risk Breast Cancer

**DOI:** 10.36959/739/525

**Published:** 2020-05-02

**Authors:** Gagan Gupta, Caroline Dasom Lee, Mary L Guye, Robert E Van Sciver, Michael P Lee, Alex C Lafever, Anthony Pang, Angela M Tang-Tan, Janet S Winston, Billur Samli, Rick J Jansen, Richard A Hoefer, Amy H Tang

**Affiliations:** 1 Department of Microbiology and Molecular Cell Biology, Leroy T. Canoles Jr. Cancer Research Center, Eastern Virginia Medical School, USA; 2 Sentara Surgery Specialists, Sentara CarePlex Hospital, USA; 3 Sentara Cancer Network, Sentara Hospital Systems, USA; 4 Department of Molecular and Cell Biology, University of California, USA; 5 Department of Pathology, Pathology Sciences Medical Group, Sentara Norfolk General Hospital, USA; 6 Department of Public Health, North Dakota State University, USA; 7 Dorothy G. Hoefer Foundation, Sentara CarePlex Hospital, USA

**Keywords:** High-risk breast cancer, Locally advanced breast cancer, Chemo-resistant breast cancer, Relapsed and metastatic breast cancer, Neoadjuvant chemotherapy, Tumor-driving RAS/SIAH/EGFR/ HER2 signaling pathways in breast cancer, Clinicopathological predictors, Therapy-responsive and prognostic biomarker for future clinical application

## Abstract

Chemo-resistant breast cancer is a major barrier to curative treatment for a significant number of women with breast cancer. Neoadjuvant chemotherapy (NACT) is standard first- line treatment for most women diagnosed with high-risk TNBC, HER2+, and locally advanced ER+ breast cancer. Current clinical prognostic tools evaluate four clinicopathological factors: Tumor size, LN status, pathological stage, and tumor molecular subtype. However, many similarly treated patients with identical residual cancer burden (RCB) following NACT experience distinctly different tumor relapse rates, clinical outcomes and survival. This problem is particularly apparent for incomplete responders with a high-risk RCB classification following NACT. Therefore, there is a pressing need to identify new prognostic and predictive biomarkers, and develop novel curative therapies to augment current standard of care (SOC) treatment regimens to save more lives. Here, we will discuss these unmet needs and clinical challenges that stand in the way of precision medicine and personalized cancer therapy.

## Unmet Clinical Need

Breast cancer is the second leading cause of cancer-relat-ed deaths in American women. Locally advanced and metastatic breast cancer is a highly heterogeneous, rapidly evolve-ing and therapy-refractory disease that challenges our ability to find curative therapies. An estimated 276,480 new cases of female breast cancer will be diagnosed and 42,170 women are expected to succumb to their malignant diseases in 2020 alone [1]. From 1989 to 2017, the death rate of female breast cancer dropped by 40% and the 5-year survival for female breast cancer between 2009 and 2015 was 90% [1–3]. These advances have been attributed to improvements in cancer prevention, increased cancer screening, and advance-es in early detection, risk stratification, anti-HER2 therapy, anti-ER therapy, anti-PI3K and anti-mTOR therapy, anti-PD1 immunotherapy, whole genome sequencing (WGS), whole exome sequencing (WES) and combination therapies [4–11].

Despite these clinical advancements, the prognosis for patients with high-grade, locally advanced, and metastatic breast cancers remains poor with an average survival of less than two or three years [2,12–14]. The decline in breast cancer mortality has slowed as the annual percent change in mortality rates for female breast cancer peaked between 1995–1998 at −3.3% but dropped to −1.5% from 2008–2017 [1]. Importantly, approximately 30% of breast cancer patients who achieved remission post first-line locoregional and systemic treatments developed recurrence in follow-up [15]. In a study of 4,105 patients in the International Breast Cancer Study Group clinical trials I to V, the annualized risk of recurrence was highest within 5-years of a diagnosis of operable breast cancer [15]. Early identification of patients at high risk of developing early tumor relapse using genomics and other multi-Omics tools has been noted to be a top priority of the Alliance Breast Committee in 2016 [5].

The clinical reality is that despite having similar clinical presentations at the time of initial diagnoses, breast cancer patients often display diverse and disparate tumor responses to standard therapies. The intrinsic diversity and evolving heterogeneity of mammary tumors can become more pronounced in locally advanced, relapsed or chemo-resistant malignant tumors post SOC therapies. This *de novo* and ac-quired tumor heterogeneity leads to diverse tumor responses to neoadjuvant and/or adjuvant systemic therapies, which in turn leads to varied clinical outcomes and disparities in patient survival [12,16,17].

## Current Prognostic Biomarkers Used in Clinical Settings

Clinicopathological parameters such as patient age, TNM (tumor size, lymph node status, metastasis) staging, tumor grade and histology, and molecular subtype of breast tumors have become commonplace in justifying medical decision making and prescribing treatment modalities. Standard radiographic assessment of newly diagnosed breast cancer involves high-resolution imaging tools including 3D digital mammography, ultrasound, MRI, and sometimes CT or PET/ CT. Traditional histopathological analysis of tumor staging in-cludes evaluating tumor size, lymph node status, and molecular sub typing using tissue markers such as estrogen receptor (ER), progesterone receptor (PR), and human epidermal growth factor (HER2) [18]. The clinicopathological parameters and ER, PR, and HER2 receptor expression status guide treatment options for breast cancer patients in each molecular subtype. All of these assessment tools hold synergistic prognostic value in deducing the clinical outcome for breast cancer patients.

The National Comprehensive Cancer Network (NCCN) clinical practice guidelines have been widely used by clinicians to treat breast cancer [19,20]. The varying levels and combination of ER, PR, HER2, and Ki67 expression characterize breast cancer patients into five intrinsic molecular subtypes [21–23]. The different categories include: luminal A (ER^+^/PR^+^, HER2^-^, Ki67^-^), luminal B (ER^+^/PR^+^, HER2^-^ or HER2^+^, Ki67^+^), HER2-over-expression (ER^-^/PR^-^, HER2^+^), basal (ER^-^/PR^-^, HER2^-^, positive basal marker), and normal-like (ER^+^/PR^+^, HER2^-^, Ki67^-^). The prevalence of luminal A, luminal B, HER2-overexpression, bas-al, and normal-like is 23.7%, 52.8%, 11.2%, 12.3% and 7.8%, respectively [24,25]. Breast cancer patients with a luminal A subtype tend to have the best outcome with a 5-year overall survival of 95.1%. Patients with the worst overall survival are triple negative breast cancer (TNBC) patients, predominately of the basal subtype. These TNBC patients have a 5-year overall survival of 75–78.5% [26–29].

The state-of-the-art high-resolution imaging modalities provide detailed information regarding the tumor size and morphology, precise spreading patterns, and clinical outcomes. Patients with breast cancer detected by mammograms have an improved disease-specific survival compared to patients whose breast cancer was detected by another imaging method, regardless of a stage shift bias [30]. Tumor staging by the American Joint Committee on Cancer (AJCC) based on Tumor Size, Node, Meta stasis (TNM) system provides prognostic estimates for the 5-year survival rate. Spe-cifically, the 5-year survival rates for patients with Stage I, IIA, IIB, IIIA, IIIB, IV disease are 95%, 85%, 70%, 52%, 48% and 18%, respectively [31]. Therefore, breast cancer patients with the poorest outcomes tend to have locally advanced tumors with local and distant metastases.

Breast cancer exhibits high inter- and intra-tumor hetero-geneity, which is especially pronounced in chemo-refracto-ry and high-grade lesions [32–36]. Tumor heterogeneity has been documented in many locally advanced, chemo-resis-tant, relapsed, high-grade and late-stage mammary tumors [32,33,36]. Tumor heterogeneity refers to the different cellular morphologic and phenotypic characteristics present in the cancerous lesion. Heterogeneity may exist within the primary tumor, between different metastatic lesions, within anoli-gometastases or between patients. As a result, this makes it difficult to treat locally advanced and metastatic breast cancer patients. Standard of care (SOC) therapy de bulks tumor masses by eliminating the drug-sensitive tumor clones; however, if the tumor eradication is incomplete, it may allow the residual drug-resistant tumor clones to continue to prolifer-ate, expand, spread, and metastasize. This may manifest with tumor recurrence and incurable disease [16,37]. The clinical reality is that current state-of-the-art therapeutic treatment modalities, alone or in combination, may not be effective in controlling and eliminating multidrug-resistant, relapsed, invasive, and metastatic breast cancer, resulting in approximately 42,000 breast cancer deaths in the U.S. every year. Therefore, a major priority in cancer medicine is the development of effective treatments to combat the prominent intra-and inter-tumor heterogeneity observed in locally advanced, relapsed, high-grade, chemo-resistant, and late-stage mammary tumors.

Currently, there are multiple different types of genomic-and transcriptomic-based techniques that are being developed to predict tumor recurrence in ductal carcinoma in situ (DCIS) and in early-stage breast cancer following SOC treatment regimens [38]. The American Society of Clinical Oncology (ASCO) recognizes the clinical utility of Recurrence Score, OncotypeDX, EndoPredict, Predictor Analysis of Microarray 50 (PAM50), Amsterdam 70-gene profile (MammaPrint), and Breast Cancer Index [39–41]. Most of these multigene assays are recommended for patients with early-stage breast cancers only; none of these gene-based assays were prognostic for patients with HER2-positive or triple-negative breast cancers independent of pathological stage [38,42,43]. The development of these promising gene signature-based molecular assessment tools for early-stage breast cancer is encourage-ing; however, there still remains an unmet clinical need to treat high-risk and high-grade patients diagnosed with locally advanced, relapsed, and metastatic breast cancers.

The Oncotype DX 21-gene Recurrence Score (RS) was developed in order to determine the likelihood of distant recurrence of early-stage breast cancer patients who initially were diagnosed with node-negative, ER^+^ luminal type breast cancer treated with tamoxifen [44,45]. The RS stratifies the risk of distant recurrence based on an algorithm of the expression levels of 16 cancer-related genes and 5 reference genes. It then scores and categorizes patients into groups of low-risk (RS < 18), intermediate-risk (18 ≤ RS < 31), or high-risk (RS ≥ 31), with a 10-year recurrence probability at 6.8% (low-risk group), 14.3% (intermediate-risk group), and 30.5% (high-risk group), respectively. Additionally, the RS was found to be sig-nificantly correlated to the relapse-free interval and overall survival [46].

Multiple prospective studies have affirmed the ability of the RS to guide clinical treatment; the TAILORx trial determined that hormone receptor (HR)^+^, HER2^-^, and axillary node-negative breast cancer patients who had a low RS ≤ 10 and were treated with endocrine therapy alone had low rates of recurrence after 5-years [47–49]. The study found that at 5-years, the rate of invasive disease-free survival was 93.8%, the rate of overall survival was 98%, and the rate of recurrence-free survival at a distant site was 99.3% [49]. Therefore, patients with low RS should avoid overtreatment, as their risk for 5-year distant recurrence is 0.7% with an RS ≤ 10.

For patients with a midrange RS score between 11 to 25, the RS may guide treatment for early-stage luminal type breast cancer. The TAILORx trial studied the outcomes of patients with low to midrange RS scores (11 ≤ RS < 25), who were treated with endocrine therapy only or with chemo-endocrine therapy in combination. The study found that there was no inferiority of endocrine therapy when compared to chemo-endocrine therapy. The endocrine therapy group and chemo-endocrine therapy group had statistically similar 9-year invasive disease-free survival (83.3% and 84.3%, respectively), freedom from disease recurrence at a distant site (94.5% and 95%, respectively), and overall survival (93.9% and 93.8%, respectively). As a result, the study concluded that the addition of chemotherapy does not improve the clinical outcome and overall survival for patients with early-stage luminal type breast cancer with a midrange RS. However, there was some benefit of the addition of chemotherapy for women under 50-years of age with an RS between 16 and 25 [50]. From this, the ASCO updated clinical guidance rec-ommends that patients older than 50 with a RS < 26 and patients 50-years old or younger with an RS < 16 should only be treated with endocrine therapy, as there is no statistically significant benefit conferred by the addition of chemotherapy on their outcomes and survival [51].

The RS may also be used to guide treatment options for breast cancer patients stratified to have an intermediate-to high-risk score. Reanalysis of the TAILORx trial showed that patients with a RS > 25 who received chemotherapy and tamoxifen had a 10-year distant recurrence-free rate at 88%, compared to an estimated 10-year distant recurrence-free rate at 62% with tamoxifen alone. Therefore, the RS may be used to identify intermediate- to high-risk patients that may benefit from the addition of chemotherapy to tamoxifen [52].

The MammaPrint assay was developed after comparing the gene expression profiles of primary breast cancer patients under 55-years old with negative lymph nodes who either did or did not have distant metastasis within 5-years [53,54]. From this analysis, the expression pattern of 70-genes was determined to be prognostic of breast cancer patients who are at low-or high-risk of recurrence. As a result, MammaPrint offers an opportunity to identify patients with a low-risk of metastasis who may benefit from avoiding unnecessary adjuvant chemotherapy, and patients with a high-risk of metastasis who would benefit from adjuvant chemotherapy [53,54].

The MINDACT trial was the first prospective study to test the clinical utility of MammaPrint. The women enrolled all had primary invasive breast cancer with involvement of three or fewer axillary lymph nodes [55]. The study assessed its patient population’s genomic MammaPrint risk and clinical risk using a modified version of Adjuvant! Online. Patients were then randomized to receive either endocrine therapy or chemo-endocrine therapy, based on their high clinical risk and low genomic risk stratification. The study found that patients with a high clinical risk and low genomic risk for recurrence who received endocrine therapy had a 5-year survival rate without distant metastasis at 94.4%. This was compared to a rate of 95.9% for patients with a high clinical risk and low genomic risk who received chemo-endocrine therapy. The survival difference between the adjuvant chemo-endocrine versus endocrine therapy is quite small at 1.5%. Therefore, it is concluded that many low-risk patients may benefit from de-escalation of chemotherapy or the withholding of chemotherapy [53,54]. However, it also may be argued that there is a benefit to the addition of chemotherapy for a subset of the high-risk patients.

From the MINDACT trial, ASCO made recommendations for the clinical use of MammaPrint to guide decision making regarding early-stage patients in the low-risk group, including ER^+^/PR^+^, HER2^-^ and node-negative breast cancers, as they may benefit from the withholding of adjuvant chemotherapy. For patients who have high clinical risk and are ER^+^/PR^+^, HER2^-^, node positive (one to three positive nodes), MammaPrint may in-form decision making on withholding adjuvant chemotherapy as well. However, it is important to reiterate the possible benefit in 5-year survival without distant metastasis with the addition of chemotherapy in this group, even though the benefit may be small. The use of MammaPrint is not recommended for high-risk patients diagnosed with HER2^+^ breast cancer or TNBC [51,56].

Other recurrence prognosis tests like EndoPredict, Breast Cancer Index, and PAM50 are similar to the Oncotype DX RS and MammaPrint. Each of the tests measures and quantifies the expression of genes of interest to determine the risk of distant recurrence. Overall, these genomic and transcriptomic analyses provide a powerful tool to stratify the risk of recurrence in early-stage breast cancer patients. However, there are several limitations to these techniques in the spec-ificity and sensitivity of gene expression profiles, dynamic range, tumor purity, and tumor/TME composition. A major limitation is that these gene expression-based prognostic and predictive tools are not effective for risk stratification in locally advanced, high-grade, relapsed, and metastatic settings, where a timely intervention and the formulation of the correct sequence of aggressive therapies are urgently needed. For instance, an RS is limited to patients with ER^+^, HER2^-^ and node-negative breast cancer, and only half of all breast cancer patients qualify for RS analysis [48]. Patients outside of these parameters include the 10% of breast cancer patients with high-risk and high-grade diseases who are known to develop tumor recurrence, resistance to therapy, and local and distant metastasis, despite receiving aggressive locoregional and systemic therapy. Additionally, ~6% of the midrange RS breast cancer population do not survive 9-years from time of diagnosis, and ~5% of the midrange RS breast cancer patients have distant recurrence within 9-years regardless of treatment with endocrine or chemo-endocrine therapy [48]. For MammaPrint, there is a 1.5% small improvement in the 5-year survival without distant recurrence for high clinical risk and low genomic risk patients who received chemo-endocrine therapy compared with those who received endocrine therapy alone. Therefore, more precise techniques are needed in order to distinctly identify this specific cohort of the at-risk patient population so that their outcomes may be improved. These limitations in RS and MammaPrint prognosis highlight the need to develop more accurate and high-preci-sion tools to identify the highest-risk patients with the aim of developing novel targeted therapies which minimize tumor recurrence and metastatic dissemination and thus, improve long-term survival in the clinic.

For some patients with advanced breast cancer, there are new treatment options being developed to treat the drug-re-sistant tumor clones. For example, alpelisib is an α-specific PI3K inhibitor that has shown great promise in the treatment of advanced breast cancer containing a *PIK3CA* mutation. The SOLAR-1 trial studied the effects of alpelisib therapy on progression-free survival of patients with HR^+^, HER2^−^, *PIK3CA* mutated, advanced breast cancer who had received endocrine therapy in the past. It found that patients who received alpelisib and fulvestrant had a progression-free survival of 11 months compared to 5.7 months for the patients who received a placebo and fulvestrant [57]. As a result, in 2017 the FDA approved the clinical use of alpelisib and Foundation One Cdx (F1CDx) as a companion diagnosis tool to guide targeted therapies. F1CDx is an *in vitro* next-generation sequencing di-agnostic tool that provides information about genomic mutations in 324 genes, gene rearrangements, microsatellite insta-bility, and tumor mutational burden (2018). Therefore, treatment-refractory HR^+^, HER2^-^, advanced breast cancer patients may undergo F1CDx analysis to determine if they possess a *PIK3CA* mutation and therefore may benefit from alpelisib treatment. With the increased clinical utility of WGS and WES data, new tumor vulnerabilities and actionable targets will be identified. New clinical trials and novel drug combination therapies will need to be deployed to treat the deadly, che-mo-resistant, and malignant tumors that kill 42,000 American women per year in the United States alone. Ultimately, to address this pressing unmet clinical need, we propose to develop a new and potent targeted therapy to control and conquer genetically diverse, heterogeneous, multidrug-resis-tant, relapsed, and metastatic breast cancer [58,59].

## Pathological Response as a Clinical Biomarker of Prognosis

Aside from genomic and transcriptomic analysis, clinical and pathological assessment following neoadjuvant therapy may be the most reliable prognostic predictors of clinical outcomes. Neoadjuvant chemotherapy (NACT) has become a standard treatment for women with high-risk TNBC, HER2^+^, and locally advanced ER+ breast cancer of mixed molecular subtypes. Following neoadjuvant therapy and surgical resec-tion of the cancerous lesion, pathological analysis of the residual tumor may provide critical insight into the risk of tumor progression and early relapse. After completion of NACT and surgery, patients fall into two groups: Those with a pathologic complete response (pCR) or pathologic incomplete response (pIR). pCR is a reliable clinical prognostic biomarker that is associated with improved outcomes and prolonged survival. Conversely, those pIR patients with chemo-resistant and locally advanced residual diseases predict a higher risk of early tumor relapse. As such, many high-risk pIR patients are now commonly considered for additional adjuvant therapies.

pCR at the primary tumor and axillary lymph nodes is associated with improved long-term survival (disease-free survival and overall survival). Moreover, it has the highest prognostic value in patients with aggressive breast cancers like TNBC [55]. pCR was associated with a better disease-free survival and overall survival than pIR regardless of *BRCA1/2* mutation status in the ERNEST-B trial comprised of breast cancer patients treated mainly with neoadjuvant anthracycline therapy [60].

Unfortunately, most high-risk and high-grade patients have residual disease after receiving NACT. Incomplete responders can be further risk stratified by pathological staging systems like calculating the Residual Cancer Burden (RCB) or the American Joint Committee on Cancer Staging post-neoadjuvant therapy (yAJCC). The RCB assesses the proportion of the residual tumor bed that contains invasive carcinoma excluding *in situ* disease, dimensions of the residual cancer in the tumor bed, the number of lymph nodes positive for residual tumor cells, and the longest diameter of the largest residual nodal metastasis. These RCB risk factors are convert-ed into a score between 0 and 3 where 0 represents a pCR tumor that has achieved a complete tumor eradication, and 3 represents the highest-risk pIR tumor that is likely to develop early tumor relapse and distant metastasis post-NACT. Some limitations to this method include the subjectivity in-volved with assessing the cellularity and the dimensions of the residual tumors [61]. However, RCB has been determined to be prognostic for 10-year relapse-free survival rates for multiple patient cohorts treated with different NACTs include-ing fluorouracil, doxorubicin, and cyclophosphamide (FAC), paclitaxel with FAC, and trastuzumab with sequential paclitaxel and fluorouracil, epirubicin and cyclophosphamide. RCB classification was prognostic for TNBC, HR^+^/HER2^-^ and HER2^+^ phenotypes independent of other prognostic factors [62,63]. While patients with an RCB score 3 have an extremely high risk of tumor recurrence, the risk is lower but still substantial for patients with an RCB score 1–2. Analysis of the I-SPY 1 trial similarly concluded that RCB was prognostic for tumor recurrence. With recursive partitioning, TNBC or HER2^+^ patients with an RCB of 3 had the highest risk of recurrence with a 3-year recurrence free survival of only 29% [61]. The yAJCC is a revised TNM staging system to assess the pathological response by characterizing the tumor based on its size (T), lymph node involvement (N) and metastasis (M) post-NACT. A limitation with yAJCC may involve the presence of scatter foci which may alter the perceived tumor size. Analysis of the I-SPY 1 trial determined that yAJCC tumor staging was prognostic for risk of recurrence. With recursive partitioning, TNBC or HER2^+^ patients with a yAJCC score of III especially had a particularly high risk of recurrence with a 3-year recurrence-free survival of only 27% [61].

The Federal Drug Administration (FDA) has recognized the prognostic value of pCR as a biomarker in the assessment of high-risk breast cancer patients. As a result, the FDA has approved the use of pCR as a distinct endpoint post neoadjuvant breast cancer therapy. The FDA defined pCR as either the complete absence of residual invasive cancer on resected tissue biospecimen and LN-negative in all resected lymph nodes (ypT0/Tis ypN0 in AJCC staging system) or the complete absence of residual invasive cancer and DCIS in resected mammary biospecimen and all lymph nodes (ypT0 ypN0 in AJCC staging system). Although RCB has also been shown to be prognostic of tumor recurrence, the FDA has chosen to use the yAJCC as the clinical standard for defining pCR [50].

The implementation of pathological response as an endpoint to NACT has aided in developing treatment strategies for high-risk and locally advanced breast cancer patients. New trials using novel therapeutic agents have been added to standard NACT with the assessment of pCR as its primary endpoint. Several post-neoadjuvant clinical trials have added a promising new drug to augment standard adjuvant therapy in hopes of reducing recurrence of metastasis, and improving the prognosis and survival of pIR patients post-NACT.

The KATHERINE trial studied the benefits of adjuvant ado-trastuzumab emtansine (T-DM1) over adjuvant trastuzumab only in HER2^+^ breast cancer patients who achieved pIR after neoadjuvant taxane with or without anthracycline and trastuzumab. Patients who received adjuvant T-DM1 compared to adjuvant trastuzumab alone had a 50% reduced risk of recurrence of invasive breast cancer or death [64]. In the CREATE-X trial, the benefits of adding capecitabine to augment adjuvant therapy for HER2^-^ breast cancer patients were assessed. The study found that the addition of capecitabine to adjuvant chemotherapy improved patients’ 3-year and 5-year disease-free survival and overall survival. The survival benefit was especially prominent in the TNBC cohort [65].

The I-SPY2 trial studied the benefits of adding pembrolizumab to standard NACT for high-risk women with stage II or III, ERBB2-negative breast cancer [66,67]. The study found that the addition of pembrolizumab to standard therapy con-sisting of paclitaxel, doxorubicin, and cyclophosphamide improved the estimated pCR rate compared to standard therapy for ERBB2-negative, HR^+^/ERBB2-negative, and TNBC (44% vs. 17%, 30% vs. 13%, 60% vs. 22%, respectively). Additionally, patients who achieved a pCR appeared to have a substantial long-term survival benefit as patients treated with pembrolizumab and standard therapy had an event-free survival of 93% at 3-years [66,67].

Studies like the KATHERINE, CREATE-X, and I-SPY 2 trial have created an interactive experimental platform address-ing multiple unmet needs facing high-risk partial responders. By using pIR as an endpoint, these studies were able to identify correct treatment sequence and stratify breast cancer patients faced with early tumor relapse, therapy-resistance, poor prognoses, and reduced survival [68,69]. Additionally, because these studies are modifications of the current SOC therapies, these state-of-the-art treatment modalities will in-crease the pace of progress, improve the likelihood of success in precision oncology, validate new prognostic and predictive biomarkers, determine the synergy and efficacy of novel therapy, decrease the financial burden, reduce toxicity, and improve overall quality of life (QOL) issues associated with conventional systemic chemotherapies [68–70].

## SIAH as a New Therapy-Responsive and Prognostic Biomarker in Breast Cancer

Seven in absentia homologue (SIAH) is a promising therapy-responsive and prognostic biomarker that may be used in conjunction with pathological response to NACT to identify high-risk breast cancer patients [71,72]. SIAH is a highly evo-lutionarily conserved E3 ligase and an essential downstream “gatekeeper” in the EGFR/HER2/K-RAS signaling pathway [73,74]. The EGFR/HER2/RAS signaling pathway activation is responsible for uncontrolled cellular proliferation, growth, and cell dissemination in a vast majority of human cancers [59,75–78]. Even though a driver mutation of the RAS signaling pathway, oncogenic K-RAS, is present in only 5% of breast cancer patients, the EGFR/HER2/K-RAS pathway is active in a large percentage of locally advanced, chemo-resistant, relapsed, and metastatic mammary tumors [71,72]. Given that SIAH is the most conserved signaling module and the most downstream “gatekeeper” enzyme in the tumor-driving EGFR/HER2/RAS signaling pathway, SIAH^ON/OFF^ expression can serve as an ON/OFF binary code and an excellent biomarker for cellular proliferation in residual tumor cells post-NACT. Furthermore, SIAH is a new, logical, and well-positioned therapeutic target to treat multidrug-resistant and incurable breast cancer [58].

SIAH has been found to be a tumor-specific, therapy-re-sponsive, and prognostic biomarker in breast cancer [72]. In a retrospective study conducted by our group, SIAH alone or in combination with EGFR had better prognostic value in high-risk breast cancer, outperforming ER, PR, HER2, and Ki67, as a new biomarker. Moreover, SIAH’s prognostic power alone was comparable to the clinical gold standard of prognostic parameters: Lymph node metastasis, mammary tumor size, grade, stage, and molecular subtypes in combination in a 5-year study [72].

**Figure 1:**
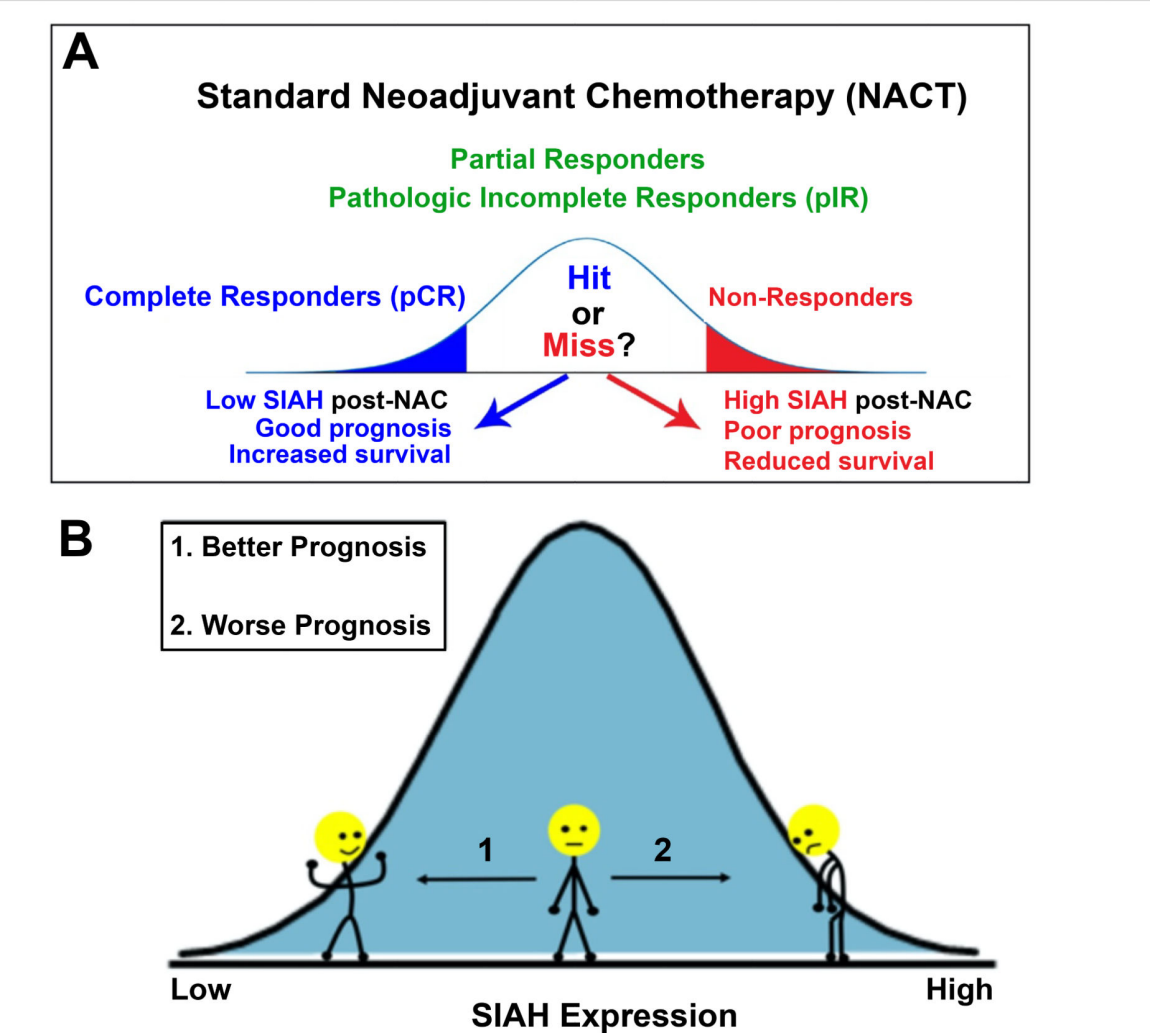
SIAH is a binary, tumor-specific, therapy-responsive, and prognostic biomarker in breast cancer. SIAH^ON/OFF^ expression in residual tumors can be used to stratify patients, identify good prognosis (SIAH expression is absent or low) or poor prognosis (SIAH expression is high), forecast early tumor relapse, predict patient survival with post-neoadjuvant chemotherapy (NACT).

SIAH expression stratified the pIR patients with residual tumors post-NACT into low-risk and high-risk groups. Partial responders with residual tumors with no or low SIAH expression (SIAH^OFF^) post-NACT are likely to remain in remission, have improved outcomes and increased disease-free survival. In contrast, partial responders with comparable RCB tumors with persistent high levels of SIAH expression (SIAH^ON^) post-NACT are likely to develop early tumor relapse, suffer poor outcomes and reduced survival (Figure 1). Given that SIAH is the most downstream and evolutionary conserved enzyme in the tumor-driving EGFR/HER2/RAS pathway, persistent high SIAH expression in the RCB tumors post-NACT correlates with continued residual tumor growth propelled by rapidly expanding chemo-resistant tumor clones, relating to tumor relapse. Assessment of SIAH expression post-NACT may become a clinically useful surrogate prognostic biomarker in quantifying therapy efficacy and tumor response post-NACT, allowing oncologists to identify chemo-resistant residual tumors, forecast early tumor relapse, and predict patient survival in real time [71,72]. Multi-centered large-scale validation studies will need to be conducted, retrospectively and prospectively, in order to quantify and incorporate the prognostic value of SIAH expression in RCB tumors as a new risk stratification factor. Furthermore, novel anti-SIAH targeted therapy will be developed to precisely treat high-risk and high-grade residual tumors with high SIAH expression that are associated with chemo-resistant, relapsed, late-stage, and metastatic breast cancers in the clinic.

## Future Perspectives

Chemo-resistant breast cancer is a major impediment to improve overall survival in breast cancer. Currently, there are no reliable clinical biomarkers to consistently guide and select SOC therapies, and accurately predict survival for patients diagnosed with high-risk, high-grade, chemo-resistant, relapsed, or metastatic breast cancers [12]. Classically utilized breast cancer biomarkers, such as ER, PR, and HER2, do not correlate with survival outcomes nor do they predict tumor response to aggressive chemotherapies in relapsed and metastatic settings [12]. Patients with locally advanced and metastatic breast cancer are often subjected to full regimens of surgical resection, chemo-and radiation therapies followed by a period of heightened anxiety and uncertainty ranging from months to years as they wait to learn the ultimate response, either tumor relapse or remission, of their dissemi-nated tumor cells post-SOC therapies. As such, there is a po-tential that some patients may be over-treated in ways that compromise their long-term QOL. Conversely, some patients may be under-or incorrectly treated and thus miss a critical window of opportunity to benefit from the lifesaving anticancer therapies.

Early stage breast cancer is highly responsive to commonly prescribed SOC therapies with excellent long-term survival. Locally advanced and metastatic breast cancer has a much worse prognosis despite aggressive chemo-and radiation therapies and loco regional surgical interventions. This dis-parity in outcomes underlines the acute need to better tailor individualized therapy and stratify patients in order to improve overall patient survival. Multidrug resistance can be in-nate or acquired, and is a leading cause of treatment failures in breast cancer. Therefore, there is a pressing need to stratify high-risk patients with tumor-specific, therapy responsive, and prognostic biomarkers, identify chemo-resistant tumor clones, monitor tumor response in real-time, improve RCB classification, and predict patient survival in partial responders post-NACT [17,79–85].

Approximately 30% of patients diagnosed with early-stage breast cancer will eventually progress to locally recurrent or metastatic breast cancer. Few therapeutic agents, alone or in combination are effective in controlling and eliminating mul-tidrug-resistant and incurable breast cancer. This results in an estimated 42,170 breast cancer deaths in the U.S. in 2020 alone. For those patients diagnosed with high-risk TNBC, HER2^+^ and locally advanced ER^+^ breast cancer, timely adminis-tration of correct, smart, personalized, precise, and effective first-line therapies is of paramount importance in saving and extending more lives.

Many improvements have been made in the diagnosis and treatment of breast cancer which has led to the 5-year survival being 90%. Unfortunately, a small subset (10%) of breast cancer patients found to have locally advanced or metastatic breast cancer have far worst outcomes. Current technology cannot conclusively distinguish whether a patient with residual disease after NACT will develop tumor recurrence or stay in remission. As such, a high priority is to accurately assess with molecular clarity and high precision which patients are at greatest risk for relapse. With the development of new genomic and transcriptomic analysis and FDA approval of pCR as an endpoint, high-risk breast cancer patients can be better identified and new therapies are being developed. The binary biomarker SIAH^ON/OFF^ is one such prognostic factor. In partic-ular, SIAH^ON/OFF^ expression offers an excellent opportunity to augment the residual tumor staging and the prognostic value of RCB, genomic and transcriptomic sequencing analyses, and pCR post-NACT. SIAH has shown promising prognostic power as a tumor-specific, tumor heterogeneity-independent, and therapy-responsive biomarker in residual mammary tumors post-NACT. Further improvements in the identification of high-risk versus low-risk partial responders may be made with the addition of SIAH and/or SIAH-interacting proteins to provide the molecular precision in patient stratification at a single tumor cell resolution. This will augment RCB risk stratification, aid the clinical decision-making process, and improve the overall survival of partial responders with high-risk residual tumors in the future.

## Abbreviations

AJCC: American Joint Committee on Cancer
ASCO: American Society of Clinical Oncology
DCIS: Ductal Carcinoma *In Situ*
ER: Estrogen Receptor
FDA: Federal Drug Administration
H&E: Hematoxylin and Eosin Staining
HER2: Human Epidermal Growth Factor Receptor 2
HR: Hormone Receptor
IHC: Immunohistochemistry
LN: Lymph Node
NACT: Neoadjuvant Chemotherapy
NCCN: The National Comprehensive Cancer Network
pCR: Pathological Complete Response
pIR: Pathological Incomplete Response
pNR: Pathological Non-Response
PR: Progesterone Receptor
QOL: Quality of life
RCB: Residual Cancer Burden
RS: Recurrence Score
SIAH: Human Homologues of *Drosophila* Seven-In-Absentia (SINA)
SOC: Standard of Care
TNBC: Triple-Negative Breast Cancer
WES: Whole Exome Sequencing
WGS: Whole Genome Sequencing
